# Group-based trajectory analysis of acute pain after spine surgery and risk factors for rebound pain

**DOI:** 10.3389/fmed.2022.907126

**Published:** 2022-08-22

**Authors:** Yi-Shiuan Li, Kuang-Yi Chang, Shih-Pin Lin, Ming-Chau Chang, Wen-Kuei Chang

**Affiliations:** ^1^Department of Anesthesiology, Taipei Veterans General Hospital, Taipei, Taiwan; ^2^School of Medicine, National Yang Ming Chiao Tung University, Taipei, Taiwan; ^3^Department of Orthopedics, Taipei Veterans General Hospital, Taipei, Taiwan

**Keywords:** group-based trajectory analysis, spine surgery, patient-controlled analgesia (PCA), rebound pain, multimodal analgesia (MMA)

## Abstract

**Background:**

This retrospective study was designed to explore the types of postoperative pain trajectories and their associated factors after spine surgery.

**Materials and methods:**

This study was conducted in a single medical center, and patients undergoing spine surgery with intravenous patient-controlled analgesia (IVPCA) for postoperative pain control between 2016 and 2018 were included in the analysis. Maximal pain scores were recorded daily in the first postoperative week, and group-based trajectory analysis was used to classify the variations in pain intensity over time and investigate predictors of rebound pain after the end of IVPCA. The relationships between the postoperative pain trajectories and the amount of morphine consumption or length of hospital stay (LOS) after surgery were also evaluated.

**Results:**

A total of 3761 pain scores among 547 patients were included in the analyses and two major patterns of postoperative pain trajectories were identified: Group 1 with mild pain trajectory (87.39%) and Group 2 with rebound pain trajectory (12.61%). The identified risk factors of the rebound pain trajectory were age less than 65 years (odds ratio [OR]: 1.89; 95% *CI*: 1.12–3.20), female sex (*OR*: 2.28; 95% *CI*: 1.24–4.19), and moderate to severe pain noted immediately after surgery (*OR*: 3.44; 95% *CI*: 1.65–7.15). Group 2 also tended to have more morphine consumption (*p* < 0.001) and a longer length of hospital stay (*p* < 0.001) than Group 1.

**Conclusion:**

The group-based trajectory analysis of postoperative pain provides insight into the patterns of pain resolution and helps to identify unusual courses. More aggressive pain management should be considered in patients with a higher risk for rebound pain after the end of IVPCA for spine surgery.

## Introduction

The indications for spine surgeries vary from herniated disks, spondylolisthesis, fractures, and tumors to scoliosis correction surgeries, and most of these patients need decompression and spine fusion surgeries. As the knowledge of spinal biomechanics, imaging diagnostics, and medical technology is improving over time, the complexity and diversity of spine surgery are increasing as well (1). Although these complex surgeries may benefit those suffering from spinal disease (2, 3), intense pain following the procedures, especially in the immediate and early postoperative period (4–6), often results in clinical problems such as delayed recovery induced by a reduction in patient mobility (7–9). As a result, effective postoperative pain control is of paramount importance and has been connected with better surgical outcomes (10, 11), reduced length of hospital stays (LOSs) (10, 11), lower incidence of chronic postsurgical pain (12), and decreased opioid dependence (7, 13). However, how to well control acute pain after spine surgery remains a major challenge for clinicians (1, 6, 7, 14, 15).

Intravenous patient-controlled analgesia (IVPCA) is a common and effective method to relieve acute pain after spine surgery (16–18) and it optimizes the delivery of analgesics and minimizes the interindividual variability in pharmacokinetics and pharmacodynamics (19). While some studies emphasized the importance of multimodal analgesia in spine surgery (1, 6, 14), IVPCA remains the gold standard for postoperative pain control for spine surgery worldwide (15–18). In addition, IVPCA provides better analgesia after spine surgery than conventional as-needed analgesic regimens do and improves patient satisfaction in the early postoperative days as well (20). However, moderate to severe rebound pain after the discontinuation of IVPCA was noted in other types of surgeries (21), and it is not clear whether this phenomenon also exists in patients receiving IVPCA for pain control after spine surgery. Accordingly, we hypothesized that some patients undergoing spine surgery were at risk of having rebound pain after the end of IVPCA and that there were risk factors associated with the development of rebound pain and designed this retrospective study to investigate these issues. The group-based trajectory analysis was used to classify the variations in postoperative pain scores over time and identify patients with rebound pain after discontinuing IVPCA. The risk factors of rebound pain were also explored, and the influence of rebound pain trajectory on the total amount of IVPCA consumption and LOS after surgery were evaluated as well.

## Materials and methods

### The inclusion and exclusion criteria

This study was approved by the Institutional Review Board of Taipei Veterans General Hospital, Taipei, Taiwan (IRB-TPEVGH no. 2020-01-003AC). Written informed consent was waived and all the included patients were de-identified before analysis. We carefully reviewed the electronic medical records of patients receiving spine surgery and postoperative IVPCA for postoperative pain control in our hospital from January 2016 to December 2018 and collected all records. Those with severe postoperative complications, less than three pain assessments in the first postoperative week, IVPCA use of fewer than 48 h, age < 20 years old, staged surgery, re-operation, or missing key data, such as operation records, were excluded from the analysis.

### Anesthesia method and pain management

In this study, all patients were administered general anesthesia with fentanyl (2–3 μg/kg) followed by propofol (1–2 mg/kg) and cisatracurium (0.2 mg/kg) or rocuronium (0.8 mg/kg) for induction. After endotracheal intubation, general anesthesia was maintained using desflurane or sevoflurane with the aforementioned neuromuscular blocking agents. Toward the end of the surgery, the inhalation agent was tapered off and the residual neuromuscular block was reversed with neostigmine and atropine. All patients were transferred to our post-anesthesia care unit where an infusion pump for IVPCA was connected to the patients with a loading dose of morphine of 2–4 mg and a bolus dose of 1 mg. No adjunct analgesics, such as acetaminophen and non-steroidal anti-inflammatory drugs, were administered with IVPCA. After the discontinuation of IVPCA on the fourth postoperative day (POD 4), pain management was shifted to oral medications, including Ultracet (acetaminophen 325 mg + tramadol 37.5 mg) every 6 h and 25 mg diclofenac every 8 h as needed.

### Data collection and endpoints

After surgery, patient-reported pain scores on a numeric rating scale (NRS) from 0 to 10 for no pain to the worst pain were recorded by the nurses in charge at least one time per day. Postoperative maximal daily pain scores were collected in series and used in the trajectory analyses. Patient attributes, such as age, sex, body mass index (BMI), and comorbidities, surgical features, such as surgical time and blood loss, PCA pump settings, and LOS after surgery were collected. The primary endpoint was the patterns of postoperative pain trajectories, and the secondary endpoints were the total amount of PCA consumption and LOS after surgery.

### Statistical analysis

Group-based trajectory analysis was employed to categorize the variations in postoperative pain over time and the technical details refer to Jones et al. (22). The numbers and features of postoperative trajectories were decided by comparing the Bayesian information criteria of different models and examining the generated trajectories and estimated parameters (23, 24). Two main patterns of pain trajectories were identified, and we compared patient characteristics between the two groups with the Student’s *t*-test, the Mann–Whitney *U*–tests, or the chi-squared tests as appropriate. The relationships between the types of postoperative pain trajectories and collected variables were evaluated and presented as odds ratios (OR) with 95% confidence intervals (CI) as well. Backward model selection with an exit criterion of significance level greater than 0.05 was performed to determine the final model for the prediction of postoperative pain trajectories. In addition, a simplified risk scoring system was developed to predict a rebound pain trajectory after the discontinuation of IVPCA for spine surgery. The area under the receiver operating characteristic (ROC) curve (AUC) was used to assess the predictive power of the final model and the simplified risk scoring system. Besides, linear backward regression analysis with an exit significance level of 0.05 was used to select independent predictors of total morphine consumption and log-transformed LOS after surgery. The adjusted association between the types of pain trajectories and total morphine consumption or LOS was also evaluated after the final predictive models were determined. A *p*-value less than 0.05 was considered statistically significant in this study. All the analyses were conducted using SAS software, version 9.4 (SAS Institute Inc., Cary, NC, United States).

## Results

### Analysis of postoperative pain trajectories

**FIGURE 1:**
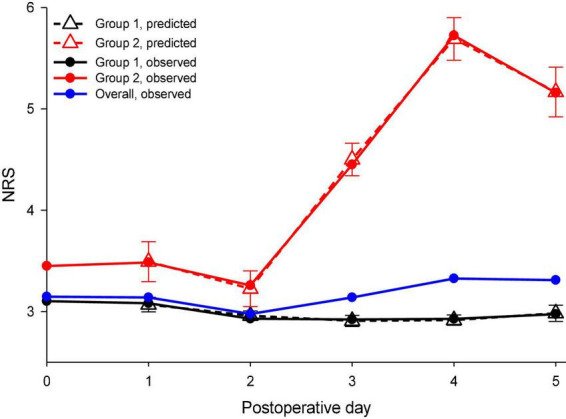
Observed and predicted maximal daily pain scores during the first postoperative week were stratified by distinct pain trajectories after spine surgery. NRS, a numeric rating scale for pain intensity. *Solid blue line*: observed overall pain scores during the first postoperative week; *solid black line*: observed pain scores of the mild pain trajectory group; *solid red line*: observed pain scores of the rebound pain trajectory group; dashed black line: predicted pain scores with their 95% confidence interval (*CI*) for the mild pain trajectory group; and *dashed red line*: predicted pain scores with their 95% confidence interval for the rebound pain trajectory group.

There were 547 patients with a pain score of 3,761 included in the analysis, and the average maximal pain scores on the first five PODs ranged between 2.98 and 3.33 (Figure 1, blue line). The mean morphine consumption was 52.6 mg and the median LOS was 7 days. The two postoperative pain trajectory groups were identified after the analysis: Group 1 with a mild pain trajectory (87.4%) and Group 2 (12.6%) with a rebound pain trajectory after the end of IVPCA (Figure 1, black line and red line, respectively). Table 1 shows the comparisons of patient attributes and no significant differences in the surgical features were found between the two groups. However, significant differences in the distributions of age, sex, and body weight were noted between those with rebound pain and their counterparts without it. Moreover, patients in Group 2 also tended to have more morphine consumption and longer LOS after surgery (both *p* < 0.001).

**TABLE 1:** Comparisons of patient characteristics between the two postoperative pain trajectory groups after spine surgery.

	Group 1	Group 2	
	(*n* = 478)	(*n* = 69)	*p*
Age ≤ 65 years	189 (39.5%)	38 (55.1%)	0.011
Sex (women)	291 (60.9%)	52 (75.4%)	0.013
Height (cm)	157.8 ± 9.1	155.9 ± 8.4	0.096
Weight (kg)	67.3 ± 14.6	63.7 ± 11.8	0.045
Body mass index ≥ 25 kg/m^2^	303 (63.4%)	42 (60.9%)	0.390
ASA physical status ≥ 3	155 (32.4%)	21 (30.4%)	0.428
Creatinine (mg/dl)	0.89 (0.77–1.07)	0.84 (0.73–1.13)	0.388
Maximal NRS before surgery	2.69 ± 1.02	2.86 ± 1.25	0.230
Surgical time > 3.5 h	235 (49.2%)	39 (56.5%)	0.155
Surgical blood loss ≥ 500 ml	219 (45.8%)	29 (42.0%)	0.323
Spine segment involved	3 (2–4)	3 (2–4)	0.503
Instrumentation	411 (86.0%)	60 (87.0%)	0.501
Spine involved			
Thoracic	49 (10.3%)	11 (15.9%)	0.116
Lumbar	458 (95.8%)	63 (91.3%)	0.095
Sacral	121 (25.3%)	16 (23.2%)	0.415
Total IVPCA consumption (ml)	50.13 ± 26.52	69.50 ± 42.55	< 0.001
Length of hospital stay days	7 (6–9)	8 (8–12)	< 0.001

Values are mean + SD, count (%) or median (IQR).

IVPCA, intravenous patient-controlled analgesia; ASA, American Society of Anesthesiologists; NRS, a numeric rating scale for pain intensity.

### Factors associated with rebound pain trajectory after the end of intravenous patient-controlled analgesia

After the group-based trajectory analysis, we identified three factors associated with the rebound pain trajectory, such as age ≤ 65 years (adjusted *OR*: 1.89, 95%, *CI*: 1.12–3.20), female sex (*OR*: 2.28, 95% *CI*: 1.24–4.19), and moderate to severe pain (NRS ≥ 4) on POD 0 (*OR*: 3.44, 95% *CI*: 1.65–7.15; Table 2). Surgical features and other patient characteristics were not related to the rebound pain trajectory. Moreover, a simplified risk scoring system for predicting rebound pain trajectory after the discontinuation of IVPCA could be developed as the following formula:


Risk score= 1*(age≤ 65years= 1,> 65= 0)+ 1*

(female= 1,male= 0)+ 2*(Moderate to severe pain on 

POD 0= 1, no to mild pain= 0)

**TABLE 2:** Risk factors of rebound pain trajectory after the discontinuation of IVPCA following spine surgery.

	β	SE (β)	OR	95% CI	*p*	Simplified risk score
Age ≤ 65 vs. > 65	0.64	0.27	1.89	1.12∼3.20	0.018	1
Sex (women vs. men)	0.82	0.31	2.28	1.24∼4.19	0.008	1
NRS ≥ 4 on POD 0	1.23	0.37	3.44	1.65∼7.15	0.001	2

OR, odds ratio; CI, confidence interval; NRS, a numeric rating scale for pain intensity after surgery; POD, postoperative day.

**FIGURE 2:**
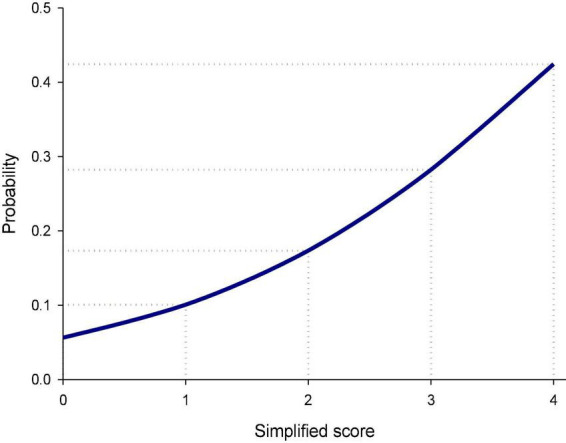
Predicted probability of the rebound pain trajectory for the simplified risk scoring systems after the discontinuation of intravenous patient-controlled analgesia (IVPCA) for spine surgery. The probability of developing rebound pain after the end of IVPCA for spine surgery increased gradually from 5.6% for the simplified score of 0–42.4% for the score of 4.

**FIGURE 3:**
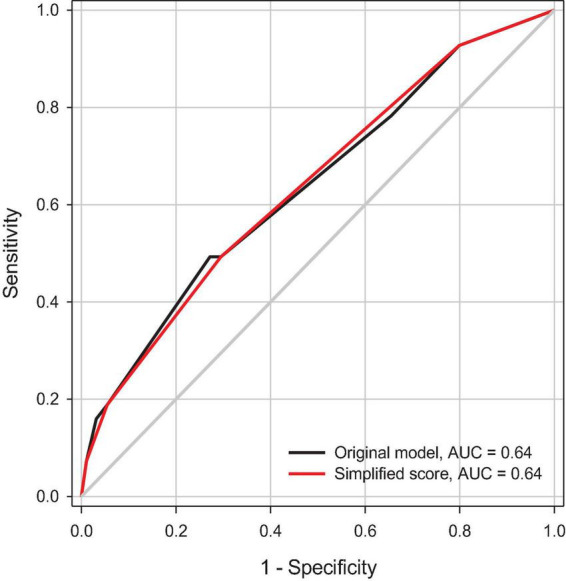
A receiver operating characteristic (ROC) curve analysis of predictive power for the selected model and the simplified risk scoring system for the rebound pain trajectory. AUC, area under ROC curve.

Figure 2 illustrates the estimated probabilities of rebound pain trajectory at distinct risk scores. The risk of developing rebound pain after the end of IVPCA ranged from 5.6 to 42.4% for patients with no to all three risk factors. Figure 3 depicts the ROC curves of the original model and a simplified scoring system. The predictive power of the two models assessed by areas under ROC curves was similar (0.64).

### Predictors of total morphine consumption after surgery

After the backward model selection processes, five independent predictors of increased morphine consumption were identified, such as age ≤ 65, male sex (both *p* < 0.001), greater preoperative pain (*p* = 0.001), more spine segment involved (*p* = 0.009), and rebound pain trajectory (*p* < 0.001; Table 3). On average, those with the rebound pain trajectory consumed 17.9 mg more morphine during their IVPCA course after controlling for the effects of other predictors in the final model.

**TABLE 3:** Predictors of total IVPCA consumption after spine surgery.

	β	SE	Std β	*p*
Pain trajectory (Group 2 vs. Group 1)	17.93	3.59	0.20	< 0.001
Age (≤65 vs. > 65 years)	13.36	2.45	–0.22	< 0.001
Sex (women vs. men)	–9.61	2.46	–0.16	< 0.001
NRS on POD 0	3.64	1.12	0.13	0.001
Spine segment involved	2.42	0.92	0.11	0.009
*Constant*	33.45	4.81	−	< 0.001

β, regression coefficients; SE, standard error; std β, standardized regression coefficients; Group 1, mild pain trajectory; Group 2, rebound pain trajectory; IVPCA, intravenous patient-controlled analgesia; NRS, a numeric rating scale for pain intensity after surgery; POD, postoperative day.

### Factors related to length of hospital stays after surgery

There were six factors associated with LOS after surgery, such as surgical time, lumbar spine involved, preoperative pain (all *p* < 0.001), American Society of Anesthesiologists (ASA) physical status (*p* = 0.005), spine segment involved (*p* = 0.014), and rebound pain trajectory (Table 4). On average, patients with the postoperative rebound pain trajectory tended to stay 17.2% longer in hospital (*p* = 0.001) than those with normal pain resolution after the adjustment for the other selected predictors in the model.

**TABLE 4:** Factors associated with length of hospital stay (LOS)* after spine surgery.

	β	SE	Std β	*p*	*exp*(β)
Pain trajectory (Group 2 vs. Group 1)	0.16	0.05	0.13	0.001	1.172
Surgical time > 3.5 h	0.19	0.03	0.22	< 0.001	1.204
Lumbar spine involved	–0.42	0.08	–0.21	< 0.001	0.660
Maximal NRS before surgery	0.06	0.02	0.14	< 0.001	1.058
ASA physical status ≥3	0.10	0.04	0.11	0.005	1.106
Spine segment involved	0.03	0.01	0.10	0.014	1.034
*Constant*	2.10	0.10		< 0.001	8.144

*Length of hospital stay is log-transformed in the analysis.

β, regression coefficients; SE, standard error of regression coefficients; std β, standardized regression coefficients; exp(β), exponentiated regression coefficients; Group 1, mild pain trajectory; Group 2, rebound pain trajectory; ASA, American Society of Anesthesiologists; IVPCA, intravenous patient-controlled analgesia; NRS, a numeric rating scale for pain intensity.

## Discussion

This is the first study to describe the phenomenon of rebound pain after the discontinuation of IVPCA for spine surgery. Although Nicholson et al. (25) used “rebound pain” to describe the increase in pain score between 8 and 24 h after surgery in a patient still “receiving PCA,” this is totally different from our findings. Approximately one-eighth of the target population experienced this unpleasant journey after the end of IVPCA. With the aid of group-based trajectory analysis, patients with abnormal pain resolution after spine surgery could be recognized and the associated factors could be identified. Regional anesthesia (RA), such as short-lasting spinal anesthesia and peripheral nerve blocks, is widely used in various surgery due to effective pain relief in the early postoperative phase. However, severe pain was noted in up to 40% of patients when the RA wears off, and this phenomenon is known as “rebound pain” (26). Recently, rebound pain was also observed in patients receiving epidural analgesia for video-assisted thoracoscopic surgery (21). All these aforementioned rebound pain phenomena were developed after the transition from an effective analgesic intervention to other routine pain management. These findings highlight the importance of analgesic transition and the necessity of early identification and intervention. Our study provides important clues for clinicians to early detect high-risk patients, and thus, preventive strategies could be initiated in advance to refine the quality of postoperative care and pain management following spine surgery (27).

Several risk factors of rebound pain were identified in this study and among them, younger age (28–31) and female sex (28–30, 32) were associated with analgesic consumption. These two non-modifiable factors were identified as risk factors in rebound pain development in other studies as well (21, 27). In addition, some previous studies revealed that younger age (28–31), female sex (28–30, 32), preoperative NRS (30, 33), and the number of spine segments involvement (4, 34) were associated with higher postoperative pain scores and more analgesic consumption. Although preoperative pain has been proposed as a risk factor for inferior postoperative pain control and more morphine consumption in a previous study (33), our study demonstrated that the postoperative pain on POD 0, rather than the preoperative pain, was an independent predictor of rebound pain trajectory after the end of IVPCA for spine surgery. The discrepancy might result from the difference in outcomes of interest and study population since we focused on the rebound pain trajectory after the end of IVPCA for spine surgery instead of general pain scores observed after surgery. Since the IVPCA remains the gold standard for postoperative pain control in complex spine surgery, the prediction of rebound pain in advance is of paramount importance. In spite of the efforts which have been made to evaluate the effects of surgical time and blood loss and the complexity of the surgery, such as procedure types and the number of spine segments involved, none of these factors were significantly associated with rebound pain trajectory. A more comprehensive classification of spine surgery might be considered in future studies.

In this study, we used group-based trajectory analysis to model the variations in pain intensity over time and identify distinct patterns of postoperative pain trajectories and their associated factors. Similar to clinical decision-making, this approach directly focuses on postoperative pain observations. Patient characteristics were not involved in the group classification processes but evaluated *post hoc* to avoid untoward interference in trajectory recognition. In addition, the group-based trajectory analysis has a great advantage in handling missing data, which is commonly observed in retrospective studies (35). Furthermore, a simplified risk scoring system was developed based on the estimated results of group-based trajectory analysis. The risk of developing rebound pain after the end of IVPCA could be easily assessed with the help of this system. Among the three risk factors, moderate to severe pain noted immediately after spine surgery despite IVPCA in use should be regarded as an early sign of possible rebound pain after the transition from IVPCA to other analgesic modalities. Once moderate to severe pain is noted after surgery, more aggressive multimodal pain management should be considered to reduce the risk of rebound pain after the end of IVPCA. This scoring system has great potential to be applied in clinical practice to prevent rebound pain after the discontinuation of IVPCA (36, 37) and improve pain control quality following spine surgery. For example, a 70-year-old male patient who is satisfied with IVPCA had no to mild pain on POD 0 after spine surgery, and the simplified risk score of rebound pain is 0; while a 60-year-old female patient who has moderate to severe pain on POD 0 under IVPCA management had the simplified risk score of rebound pain of 4. The probability of developing rebound pain (group 2) after the end of IVPCA in these two patients would be 5.6 and 42.4%, respectively. The clinicians should introduce more vigorous pain management, such as prolonged PCA duration or multiple model pain management control to prevent or manage the rebound pain afterward. However, its validity and clinical utility of this risk scoring system await further investigation.

There were some limitations to our study. First, the impacts of unobserved variables on the patterns of variations in postoperative pain scores over time could not be further evaluated and more covariates should be included in future studies for better prediction of the rebound pain trajectory. Second, the preoperative analgesic prescriptions were not further investigated due to data unavailability. Third, we only evaluated the effects of surgical time, blood loss, instrumentation, and spine segments involved on the risk of having a rebound pain trajectory but did not further assess the associations between different kinds of spine surgical procedures and the incidence of rebound pain since there is still no consensus on the classification of complex spine surgery.

In conclusion, two major patterns of postoperative pain trajectories were recognized in patients receiving IVPCA for spine surgery using group-based trajectory analysis, and about one-eighth of them had a rebound pain trajectory. Three predictors of rebound pain trajectory were identified, namely, younger age, female sex, and moderate to severe pain on POD 0. A simplified risk scoring system was developed based on the analytical results but its clinical utility needs further investigation. Preventive strategies, such as early introduction of more aggressive multimodal analgesia, should be considered in high-risk patients to reduce the incidence of rebound pain since patients with rebound pain trajectory were inclined to have longer hospital stay after surgery and more opioid consumption. Group-based trajectory analysis provides valuable information to categorize variations in postoperative pain over time and detect unusual patterns of pain resolution for further optimization of perioperative pain management. More patient attributes and surgical features should be collected in future studies to further elucidate the underlying mechanism of rebound pain after the end of IVPCA for spine surgery.

## Data availability statement

The raw data supporting the conclusions of this article will be made available by the authors, without undue reservation.

## Ethics statement

The studies involving human participants were reviewed and approved by Institutional Review Board of Taipei Veterans General Hospital. Written informed consent for participation was not required for this study in accordance with the national legislation and the institutional requirements.

## Author contributions

Y-SL contributed to data acquisition and manuscript drafting. W-KC contributed to data validation and draft preparation. S-PL contributed to study coordination and data acquisition. M-CC helped to review and revised the manuscript. K-YC contributed to conceptualization, statistical analysis, and manuscript revision. All authors have read and agreed to the published version of the manuscript.
